# Antioxidant, Antihypertensive, Hypoglycaemic and Nootropic Activity of a Polyphenolic Extract from the Halophyte Ice Plant (*Mesembryanthemum crystallinum*)

**DOI:** 10.3390/foods11111581

**Published:** 2022-05-27

**Authors:** Marta María Calvo, Ana Belén Martín-Diana, Daniel Rico, María Elvira López-Caballero, Oscar Martínez-Álvarez

**Affiliations:** 1 Institute of Food Science, Technology and Nutrition (ICTAN-CSIC), C/José Antonio Novais 10, 28040 Madrid, Spain; mmcalvo@ictan.csic.es (M.M.C.); elvira.lopez@ictan.csic.es (M.E.L.-C.); 2 Agricultural Technological Institute of Castile and León (ITACyL), Government of Castile and León, Ctra. de Burgos Km. 119, Finca Zamadueñas, 47071 Valladolid, Spain; mardiaan@itacyl.es (A.B.M.-D.); ricbarda@itacyl.es (D.R.)

**Keywords:** ice plant, polyphenols, total antioxidant activity, angiotensin-converting enzyme, prolyl oligopeptidase, dipeptidyl peptidase IV

## Abstract

This study aims to determine the potential antioxidant, antihypertensive, hypoglycaemic and nootropic activity of a purified polyphenolic extract from the halophyte ice plant (*Mesembryanthemum crystallinum*). The ice plant extract showed good antioxidant activity measured by DPPH, ORAC, TEAC, FRAP and ferrous ion chelating activity. Moreover, the extract showed potent ACE, DPP-IV and PEP-inhibitory activity (90.5%, 98.6% and 73.1%, respectively, at a final concentration of 1 mg/mL). The extract was fractionated and the fraction with the highest content of total phenolic compounds showed the highest bioactivity, suggesting that polyphenols could be mainly responsible for the abovementioned activities. The tentative polyphenol identification by HPLC-ESI-QTOF-MS in this fraction revealed that flavones (>65%) are the major group, with apigenin (38%) predominating, followed by diosmin (17.7%) and luteolin (11.9%). They could presumably be the main elements responsible for the enzymatic inhibition activity. Additionally, 4-hydroxybenzoic acid, p-coumaric acid and a hydroxycinnamic acid derivative (2-O-(p-cumaroyl)-l-malic acid) were found in the extract. To our knowledge, this is the first time that some of these activities have been reported for halophyte extracts.

## 1. Introduction

In developed countries, chronic diseases such as heart disease, stroke, cancer, diabetes and arthritis are the leading causes of death and disability [1]. All these pathologies have an important social and economic impact on society according to the World Health Organization (WHO). Many studies report the role of antioxidants in the reduction in chronic diseases and how the decrease in the intake of these antioxidants and the increase in thiobarbituric acid reactive substances favor some types of chronic pathologies. Antioxidants are of great interest for their positive effects against oxidative stress. Among them, polyphenols constitute the largest category of compounds within the secondary metabolism of plants. Their synthesis is derived from the shikimate pathway and is induced in response to unfavorable environmental conditions, which increase the generation of radical oxygen species (ROS). In this sense, a high content of polyphenols has been described in halophyte plants as a response to their growth in soils with high salinity and, sometimes, in areas with high UV radiation and sudden thermal changes [2].

Hypertension is one of the major risk factors for cardiovascular disease. In the physiological regulation of blood pressure, angiotensin I converting enzyme (ACE), a dipeptidyl carboxypeptidase, catalyzes the formation of vasoconstrictor Angiotensin II from Angiotensin I. Likewise, this enzyme is responsible for the inactivation of bradykinin, preventing it from exerting a vasodilator effect. ACE has become an interesting therapeutic target and, in fact, numerous ACE inhibitors such as captopril, enalapril, alcacepril and lisinopril are currently on the market. However, the use of these drugs leads to the appearance of side effects such as cough, taste alterations, skin rashes and angioedema; for all these reasons, there is interest in finding new nutraceuticals that may be useful to control or prevent arterial hypertension. Numerous peptides derived from animal by-products have been tested as ACE inhibitors [3], but in recent years, there has been a growing interest in finding ACE inhibitors of a polyphenolic nature that also allows for the use of underutilized vegetable by-products or raw materials.

Prolyl oligopeptidase, also known as Prolyl endopeptidase (PEP), is a serine protease highly expressed in the brain and involved in learning and memorization processes. PEP acts in the maturation and degradation of peptide hormones, such as thyrotropin-releasing hormone and vasopressin. Changes in PEP expression levels and its increased activity have been correlated with aging and have been detected in many neurodegenerative diseases, such as depression, bipolar affective disorder, schizophrenia and anxiety. PEP has been identified as a pharmacological target for the management of several cognitive disorders, especially Alzheimer’s and Parkinson’s disease. PEP inhibitors, including polyphenols, have been proposed as potential drugs for the prevention and treatment of neurological diseases [4,5,6].

Dipeptidyl Peptidase IV (DPP-IV) is an enzyme implicated in glycaemia regulation. This enzyme belongs to the same family of serine proteases as PEP, and their three-dimensional structures are very similar, although their protein chains show a low degree of homology [7]. DPP-IV is responsible for the inactivation of the incretin’s glucagon-like peptide 1 (GLP-1) and gastric inhibitory polypeptide (GIP). GLP-1 and GIP are secreted in the intestine in response to enteral digestion and trigger insulin secretion in the pancreas, although GLP-1 and GIP receptors are also found in the central nervous system, heart, lungs and peripheral tissues. The incretin effect of GLP-1 is preserved in patients with type II diabetes, thus, DPP-IV inhibitors such as Vildagliptin, Sitagliptin and Saxagliptin are currently used in their treatment [8,9]. Recently, Singh et al. [9] suggested a potential use for natural antioxidants derived from plants, such as alkaloids, phenolic acid, steroids and flavonoids, as DPP-IV inhibitors. 

The ice plant is a halophilic plant belonging to the family Aizoaceae. Native to southern and eastern Africa, it is now widely distributed throughout the world. The ice plant is tolerant to low temperatures and can grow in saline soils without this affecting its biomass production or nutritional value [10]. Recently, Loconsole et al. [11] suggested the use of the wild ice plant as a saline crop, also describing its potential use in phytoremediation, human health and as food, since it is traditionally consumed in some places [12]. In addition to its succulent texture, the leaves of the ice plant are coated by epithelial bladder cells, which resemble dewdrops and burst when chewed, leaving a slightly salty taste reminiscent of the sea. This makes the ice plant highly appreciated in haute cuisine, and the interest in its consumption has spread to many other consumers. Nonetheless, it is still considered an underutilized crop. Some studies related to *M. crystallinum* refer to its antioxidant activity [13,14]; however, there are very few references in the literature demonstrating other potentially bioactive properties of extracts of this plant.

The healthy effect of polyphenols on highly prevalent diseases such as diabetes, hypertension or neurodegenerative diseases, and their high content in halophytes, suggests that some abundant and underused salt-tolerant species could be used as a source of healthy polyphenolic extracts. It would encourage their cultivation and the control of soil erosion in certain areas. In this context, the health benefits of the ice plant are well known, and its hypoglycaemic, anti-inflammatory, antiseptic and neuroprotective effects, among others, have been described [11], although the molecules responsible for these properties have hardly been studied. Therefore, this work aims to explore the bioactive potential of an ice plant extract as an antioxidant agent, as well as its antihypertensive, nootropic and hypoglycaemic potential, and to identify the polyphenols responsible for these bioactivities. This study will contribute to improving the underutilization of plants, in line with the strategy adopted by the European Commission on the new circular economy action plan (CEAP) of 2020, which aims to promote circular economy processes, encourages sustainable consumption and seeks to avoid waste and maintain the value of resources used in the EU economy for as long as possible.

## 2. Materials and Methods

### 2.1. Chemicals

For this research, 2,2-diphenyl-1-picrylhydrazyl (DPPH), 3,4-Dihydro-6-hydroxy-2,5,7,8-tetramethyl-2H-1-benzopyran-2-carboxylic acid (Trolox), fluorescein, 2,2′-diazobis-(2-aminodinopropane)-dihydrochloride (AAPH), 2,2′-azino-bis-(3-ethylbenzothiazoline-6-sulphonic acid) (ABTS), Fe(III) chloride, 2,4,6-tri (2-pyridyl)-s-triazine (TPTZ), ferrozine, Folin–Ciocalteu reagent, gallic acid (GA), angiotensin-converting enzyme from rabbit lung, human dipeptidyl peptidase-IV and reagents used in HPLC analysis were obtained from Sigma-Aldrich, Co. (St. Louis, MO, USA). The prolyl endopeptidase from Flavobacterium was from Seikagaku Corp. (Tokyo, Japan). The chromogenic substrates (Abz-Gly-Phe(NO2)-Pro) and (Z-Gly-Pro-7-amido-4-methylcoumarin) were from Bachem (Bubendorf, Switzerland). Ethylenediaminetetraacetic acid (EDTA) was from Leco Corp. (St. Joseph, MI, USA). Other chemicals and reagents of analytical grade were obtained from Panreac Chemical Co. (Barcelona, Spain). 

### 2.2. Plant Processing

The ice plant specimens were collected from the northwest coast of Spain (Galicia), kindly provided by the company Porto-Muiños S.L. (Cerceda, A Coruña, Spain) for this study. The edible parts of the plant were packed and transported under refrigeration to the laboratories. The plants were cleaned with distilled water and immediately dried at 55 °C in a forced-air oven for 24 h (FD 240, Binder, Tuttlingen, Germany). After that, the material was stored at a low temperature (4 °C) until analysis.

### 2.3. Extract Preparation

The extract was prepared according to Sánchez-Faure et al. [15]. The dried plants (250 g) were homogenized in 400 mL of ethanol/ultrapure water mixture (1/1 *v*/*v*) acidified to pH 2 with 0.1 M HCl, using a T-25 Ultra-Turrax homogenizer (25,000× *g*, 3 min, 25 °C) (Mod. T25D, IKA^®^-Werke GmbH & Co. KG, Staufen, Germany). The mixture was placed in an ice bath and sonicated with a Q700 sonicator (Qsonica, Newton, CT, USA), using 16 min cycles at 90% amplitude, with 60-s intervals every minute. The supernatant was collected after centrifugation at 12,000× *g* for 10 min at low temperature (Sorvall evolution, Thermo Fisher Scientific, Waltham, MA, USA). After evaporation of ethanol in a rotary evaporator (R-300, BÜCHI, Flawil, Switzerland), the extract was lyophilized and stored at a low temperature (4 °C) until analysis. 

### 2.4. Determination of Total Phenol Content

The determinations were performed using the Folin–Ciocalteau assay on a UV-1601 spectrophotometer model CPS-240 (Shimadzu, Kyoto, Japan). The total content of Folin reactive substances was expressed in mEq GA/g (d.m.).

### 2.5. Determination of Total Antioxidant Capacity (TAC)

TAC was measured using different classical assays, namely radical scavenging activity (DPPH), oxygen radical absorbance capacity (ORAC), Trolox equivalent antioxidant capacity (TEAC), ferric reducing antioxidant power (FRAP) and ferrous ion chelating activity.

#### 2.5.1. DPPH Radical Scavenging Activity

DPPH radical scavenging activity of the samples was determined according to Brand-Williams et al. [16], with slight modifications. Firstly, DPPH was dissolved in pure methanol at a final concentration of 100 µM. Then, 125 µL of DPPH, 25 µL of sample and 100 µL of Milli-Q water were mixed, and the decrease in absorbance at 515 nm was recorded for 30 min using a microplate reader (Fluostar Omega, BMG Ortenberg, Germany). Results were expressed as µEq Trolox/g (d.m.).

#### 2.5.2. Oxygen Radical Absorbance Capacity (ORAC)

The ORAC assay was performed according to Ou et al. [17], with modifications. Firstly, Trolox (standard) was diluted at different concentrations (15–240 mM) in working buffer (10 mM phosphate buffer, pH 7.4). The samples were also diluted in the same buffer (10 mg/mL, d.m). Then, 150 µL of fluorescein and 25 µL of sample, standard or working buffer (blank) were mixed and incubated at 37 °C for 3 min before the addition of AAPH (2,2′-Azobis(2-methylpropionamidine) dihydrochloride) solution. Fluorescence was measured at λexc = 485 nm and λem = 528 nm for 35 min using a microplate reader. The total areas under the fluorescein decay curves of blanks, standard and samples were measured to calculate the results, which were expressed as µEq Trolox/g of sample (d.m.). 

#### 2.5.3. Trolox Equivalent Antioxidant Capacity (TEAC)

A TEAC assay was performed according to Re et al. [18], with modifications. Firstly, 7 mM ABTS+ was mixed with 2.45 mM potassium persulfate in a 1:1 (*v*/*v*) ratio. The mixture was kept in the dark for 6 h at room temperature. Then, an aliquot was diluted in 75 mM phosphate buffer (pH = 7.4) to obtain a working solution with an absorbance value of 0.70 ± 0.02 at 734 nm. Twenty µL of the sample (10 mg/mL, d.m.) were mixed with 200 µL of ABTS·+ working solution, and the absorbance at 730 nm was measured after incubation for 30 min at 30 °C. Trolox was used as the standard. The results were expressed as µEq Trolox/g of sample (d.m.).

#### 2.5.4. Ferric Reducing Antioxidant Power (FRAP)

A FRAP assay was performed according to Benzie and Strain [19], with modifications. Firstly, the sample (10 mg/mL, d.m.) was dissolved in distilled water (1:1, *w*/*v*). The FRAP reagent was prepared by mixing 25 mL of 0.2 M sodium acetate buffer (pH 3.6), 2.5 mL of 10 mM TPTZ (2,4,6-Tris(2-pyridyl)-s-triazine) dissolved in hydrochloric acid (40 mM) and 2.5 mL of iron chloride solution (20 mM). Ammonium iron (II) sulfate, or Mohr’s salt, was used as a standard. Next, 30 µL of sample or standard were mixed with 90 µL of distilled water and 900 µL of FRAP solution and incubated for 40 min at 37 °C in the dark. Then, the absorbance of the supernatant at 595 nm was measured using a spectrophotometer. The results were expressed as mEq Mohr’s salt/g of sample (d.m.). 

#### 2.5.5. Ferrous Ion Chelating Activity

The sample was firstly dissolved in distilled water (20 mg of extract or 1 mg of fraction/mL). Then, 1 mL of sample, distilled water (blank) or EDTA (standard) were mixed with 3.7 mL of distilled water and 100 μL of 2 mmol/L FeCl2. After 3 min, 200 μL of 5 mM ferrozine were added and the mixture was incubated for 10 min at room temperature. Then, the absorbance at 562 nm was measured using a microplate reader. A sample control without the addition of ferrozine was also used. Results were expressed as mEq EDTA/g of sample (d.m.). 

### 2.6. Determination of ACE Inhibitory Activity

The ACE inhibitory activity was measured according to Sentandreu and Toldrá [20], with modifications. Firstly, ACE was diluted in a 150 mM Tris-base buffer, pH = 8.3, with 1.125 M NaCl, to reach an enzymatic activity of 15 mU/mL. One unit (1 U) corresponded to the amount of enzyme that releases one μmol of hippuric acid from hippuryl-His-Leu per minute at pH 8.3 and 37 °C. The extract (1 mg/mL) or the fractions (0.1 mg/mL) were diluted in the assay buffer. Then, 50 µL of sample or buffer assay (control) were mixed with 50 µL of ACE and incubated for 5 min at 37 °C. Then, 200 µL of substrate (0.45 mM Abz-Gly-Phe(NO2)-Pro, dissolved in the assay buffer) were added. The increase in fluorescence at λexc = 360 nm and λem = 400 nm was quantified for 30 min using a microplate reader, Sinergy Mx (BioTeck, Colmar, France). The inhibitory activity was determined from the maximal increase in fluorescence per minute, in the absence or presence of the sample, and expressed as a percentage of inhibition. 

### 2.7. Determination of PEP Inhibitory Activity

The PEP-inhibitory activity was determined according to Sila et al. [21]. The final concentration in each well was 1 mg of dried weight/mL (extract) or 200 µg/mL (fractions). The samples were previously diluted in 0.1 M sodium phosphate buffer pH 7 (working buffer). The enzyme was dissolved in working buffer to reach an enzymatic activity of 1 mU. It was defined by Seikagaku Corp. as the enzyme activity that gives 1 nmol of p-nitroaniline/min at 30 °C, pH 7.0, from Z-Gly-Pro-pNA. Twenty μL of PEP (1 mU) were mixed with 180 μL of assay buffer (control samples) or with 150 μL of working buffer and 30 μL of diluted sample. After 15 min at 30 °C, 100 μL of substrate (0.01 mM Z-Gly-Pro-AMC dissolved in working buffer) was added. The blanks included the enzyme previously inactivated with 5 N HCl. Fluorescence at λexc 340 nm and λem 450 nm was measured at 1 min intervals for 20 min using a microplate reader. The maximum linear increase in fluorescence/min was calculated for controls, blanks and samples to determine the inhibitory activity, which was determined and expressed as described in Section 2.6. 

### 2.8. Determination of DPP-IV Inhibitory Activity

The DPP-IV-inhibiting activity of the samples was determined as described by PEP-activity but using 0.1 M Tris-HCl (pH 8) as a standard buffer, according to Sila et al. [21], and using 0.025 mM AMC-H (H-Gly-Pro-7-amino-4-methylcoumarine) as a substrate. The results were calculated and expressed as previously explained in Section 2.6. 

### 2.9. Chromatographic Fractionation of the Extracts

Briefly, the lyophilized extract was resuspended in a mixture of ethanol/water (50/50, *w*/*v*), obtaining a concentration of 80 mg/mL. Then, 1.5 mL were injected into a C18 preparative column (Tracel Excel 120 ODS-A 25 cm × 0.78 cm, Teknokroma, Barcelona, Spain) using a preparative HPLC (Agilent LC PREP 1260 Infinity Series, Santa Clara, CA, USA) with an array diode detector. Phase A consisted of ultrapure water containing 0.1% formic acid and 5% acetonitrile, and phase B consisted of acetonitrile containing 0.1% formic acid. The gradient used was: 0–15% B for the first 15 min, 15–50% B for the next 5 min, 50–65% B for the following 15 min, and 65–0% B for the last 10 min; the flow rate was 2 mL/min. The fractions were collected using an automated fraction collector; the detection was performed at 214, 280, 360 and 530 nm. Solvents from fractions were evaporated in a Speed Vac Concentrator (Thermo Fisher, Waltham, MA, USA); after that, the fractions were freeze-dried and stored at low temperature (4 °C) until used.

### 2.10. Tentative Identification of Polyphenols by HPLC-QTOF

The characterization of the polyphenols in the fraction with the highest activity was performed by HPLC-ESI-QTOF-MS. Solid-phase extraction of polyphenols was carried out using Oasis HLB 1 cc Vac Cartridges (Waters, Milford, MA, USA). 

The separation of polyphenols was conducted as previously described by Sánchez-Faure et al. [15] using an HPLC Agilent 1200 (Agilent Technologies, Waldbronn, Germany) equipped with a diode array detector (DAD, ref. G1315B) and an ESI-QTOF-MS (Agilent G6530A). The mass spectrum was obtained by electrospray ionization in negative and positive modes. The gas temperature was 325 °C, and the drying gas flow was 12 L/min. Scans were acquired for auto MS/MS from 100 to 1200 m/z. The MassHunter Workstation software version 4.0 (Agilent Technologies) was used to analyze the mass spectra. The phenolic compounds were identified with the molecular formula proposed by the software and comparing the experimental mass with the exact mass, allowing an error of 8 ppm. The fragmentation pattern and relative abundance of fragmentation ions were compared with the fragmentation patterns found in the public databases, Human Metabolome Database and MassBank. The relative abundance of each tentatively identified compound was determined by measuring its peak area.

### 2.11. Statistical Analysis

Statistical analysis was performed by Student’s paired *t*-test using IBM SPSS STATISTICS 27 (IBM Corporation, Armonk, NY, USA), with a 95% confidence interval. All determinations were performed in triplicate.

## 3. Results and Discussion

### 3.1. Bioactivity of Extracts

#### 3.1.1. Antioxidant Activity

The most commonly used methods to determine the total antioxidant capacity assays can be divided into two major groups: those based on a single electron transfer reaction and the ones based on a hydrogen atom transfer reaction [22,23]. The first includes the TEAC, FRAP and the DPPH assays, while the second includes the ORAC assay. In this work, the ability of the extract to exert antioxidant activity through different pathways was evaluated using different methods: the DPPH, TEAC, FRAP, ORAC assays and ferrous ion chelating activity. 

The ice plant extract showed good antioxidant activity (Table 1). The value obtained for DPPH was similar to that found by Ngxabi et al. [24] in an extract of the halophytic species Trachyandra ciliate (19.01–9.39 μmol TE/g). The ice plant extract showed 88 μEq Trolox/g determined by ORAC. A lower value was found in Chritmum maritimum extracts (15.84 μmol/Trolox g) [25], while a maximum of 101 μmol/Trolox g of the fresh matter was reported for extracts of Chritmum maritimum, Triglochin maritima and Halimione portulacoides by Boestfleisch and Papenbrock [26]. However, it is important to note that these extracts were obtained using 80% methanol, which significantly affects the final antioxidant properties. The antioxidant capacity determined by TEAC showed lower values than those reported by Cybulska et al. [27] in polyphenol extracts of Salicornia sinus-persica and Salicornia bigelovii (1227 and 4795 μmol/g, respectively).

The antioxidant activity of plant extracts is associated with the presence of antioxidant enzymes and secondary metabolites, mainly phenolic compounds (phenolic acids, flavonoids and tannins) and carotenoids. The ice plant extract had a slightly higher phenol content (Table 1) than that reported for the same plant by Sánchez-Faure et al. [15], which was 8.2 mEq GA/g. Hanen et al. [28] described a much lower phenol concentration in the same plant and Mesembryanthemum nodiflorum (1.43–1.71 mEq GA/g, respectively) and a much higher one (70.07 mEq GA/g) in an extract of Mesembryanthemum edule.

The presence of antioxidant compounds in halophytes is related to their ability to cope with the accumulation of reactive oxygen species (ROS), produced by the unfavorable conditions in which they grow. These compounds could delay the processes of cellular damage, senescence processes and metabolic disorders associated with the presence of these radicals by inhibiting the initiation or propagation of the oxidative chain reaction. Therefore, the variations observed in the antioxidant capacity of different halophyte plant extracts could be related to the concentration of secondary metabolites induced by the salinity of the soil in which these plants grow, and thus, could also be associated with the different polyphenol composition of the extracts together with the presence of different concentrations of other antioxidant compounds such as vitamin C, vitamin E, alkaloids or bilirubin [26]. The antioxidant activity of polyphenols depends on factors such as the number and position of hydroxyl groups, as well as the nature of the substitutions in the aromatic rings. The antioxidant activity of halophyte extracts could even be greater than that of synthetic antioxidants, according to Ksouri et al. [29], hence the interest in these extracts as promising alternative antioxidants to the synthetic ones traditionally used by the food industry. In addition, the analyzed extract may have some beneficial health effects due to its antioxidant potential, some of which have already been described, such as the protection against UV exposure [30] and an inhibitory effect on colon cancer cells [31].

The ice plant extract showed a FRAP value in the range of that found by Qasim et al. [32] for 100 medicinal halophyte plants. The ferrous ion chelating activity of the extract (Table 1) was much lower than that reported by Sadeer et al. [33] in an extract from the halophyte *Bruguiera gymnorhiza* (6–60 mg EDTA Eq/g).

#### 3.1.2. ACE, PEP and DPP-IV Inhibitory Activity

The ice plant extract showed potent ACE-inhibitory activity, with values of 90.5% at a concentration of 1 mg/mL (Table 1). This effect could be due to the presence of phenolic compounds in the extract [34]. 

The ACE-inhibitory activity of halophyte extracts has been scarcely reported. Men et al. [35] found lower inhibitory activity (83%, 100 mg/mL) in extracts of *Suaeda physophora*. These authors attributed the ACE-inhibitory activity to the presence of polyphenols in the extracts. Some ACE-inhibiting polyphenols from other halophytes such as *Artemisia scoparia* [36] and *Salicornia ramosissima* [37] have been isolated. Sharifi et al. [38], in an in vivo study with rats, observed an antihypertensive effect of an aqueous extract of the halophyte *Tribulus terrestris* and suggested that it could be related to an ACE inhibitory effect, as the enzymatic activity was significantly reduced in all tissues. Phillips et al. [39] also found that *T. terrestris* extracts were able to exert an antihypertensive effect in spontaneously hypertensive rats, although, in this case, it was related to smooth muscle relaxation by nitric oxide release. 

In the current study, the extract also showed potent DPP-IV and PEP-inhibitory activity (Table 1). The PEP inhibitory capacity of extracts from several plants has been described and ascribed to the presence of specific peptides and cyclotides, alkaloids such as berberine and flavonoids such as oroxylin and hispidulin [7,40,41]. Nonetheless, other molecules could also be responsible for this bioactivity. As regards the potential hypoglycaemic effect of halophyte extracts, it has been scarcely reported. Benwahhoud et al. [42] observed a hypoglycaemic effect of the edible halophyte *Suaeda fruticosa* in diabetic rats. This hypoglycaemic effect, however, may not be due to the inactivation of DPP-IV, as it was not associated with changes in plasma insulin levels in the experimental animals. These authors found flavonoids in the extract, but could not identify the compound responsible for the bioactivity. The effect of polyphenols in diabetes prevention suggests that polyphenols present in the ice plant extract could be responsible for the DPP-IV inhibiting activity [43,44].

### 3.2. Fractionation of the Ice Plant Extract

**Figure 1:**
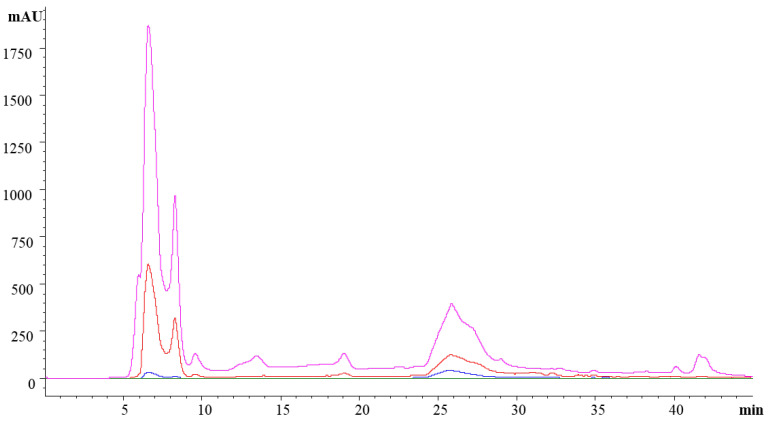
Chromatographic profiles of the ethanolic extract of ice plant. Absorbance was measured at 214 nm (pink), 280 nm (red), 360 nm (blue) and 530 nm (green). Fractions were collected at 5–10 min (fraction 1) and 24–30 min (fraction 2).

The ice plant extract was fractionated by preparative HPLC to identify the molecules responsible for the bioactive properties observed in the in vitro studies. Two major fractions were detected at the elution times of 5–10 min (called fraction 1) and 24–30 min (called fraction 2), as depicted in Figure 1. The lack of peaks at 530 nm indicated the absence of anthocyanins in the extract. Fraction 1 showed two peaks detected at 214, 280 and 360 nm. The highest was found at 214 nm, suggesting that this fraction was composed primarily of compounds with amide bonds, such as peptides, vitamins or hormones [45]. Presumably, the peaks detected at 280 nm were not only the result of the presence of aromatic amino acids in this fraction, as Sánchez Faure et al. [15] found low amounts of these amino acids in the ice plant. The low phenol content in this fraction (Table 2) suggests that these molecules were not the main ones responsible for the high absorbance found at 280 nm.

Fraction 2 showed a single peak detected at 214, 280 and 360 nm. The maximum was found at 214 nm, but the intensity was much lower than that observed in fraction 1. The high phenol content found in this fraction (Table 2) could explain the absorbance detected at 360 nm, the maximum for the flavonol group [46]. The peak was also detected at 280 nm, which is the maximum absorbance of some polyphenols. The concentration of polyphenols in both fractions was higher than that found by Hanen et al. [28] (1.43 mEq GA/g) and by Sánchez-Faure et al. (8.2 mEq GA/g) in the same plant. Differences in total phenol content could be related to different environmental factors, such as plant exposure to UV rays, soil salinity or soil drought, as well as to the extraction process and the polarity of the solvents used.

#### 3.2.1. Antioxidant Activity of the Fractions

Fraction 2 showed the highest antioxidant activity in almost all the assays performed (Table 2), which could be ascribed to the presence of highly antioxidant compounds such as polyphenols and carotenoids, as previously mentioned [47,48]. The values were much higher than those obtained for the crude extract, indicating that the main antioxidative molecules were concentrated in the second fraction. As an exception, fraction 1 showed a higher capacity to chelate ferrous ions (Table 2), which could be due to the presence of chelating peptides and free amino acids in this fraction [49].

#### 3.2.2. ACE, PEP and DPP-IV Inhibitory Activity

Fraction 2 showed significantly higher ACE, PEP and DPP-IV inhibitory activity than fraction 1 at the same concentration tested (Table 2). This suggests that the polyphenols present in fraction 2 exerted a greater inhibitory effect than the peptides and other compounds presumably present in fraction 1. Interestingly, the ACE- and PEP-inhibiting activity of fraction 2 was similar to that of the crude extract, but using a five-fold lower concentration, indicating that the main molecules responsible for the inhibition were concentrated in this fraction.

### 3.3. Identification of Polyphenols in the Active Fraction

**Figure 2:**
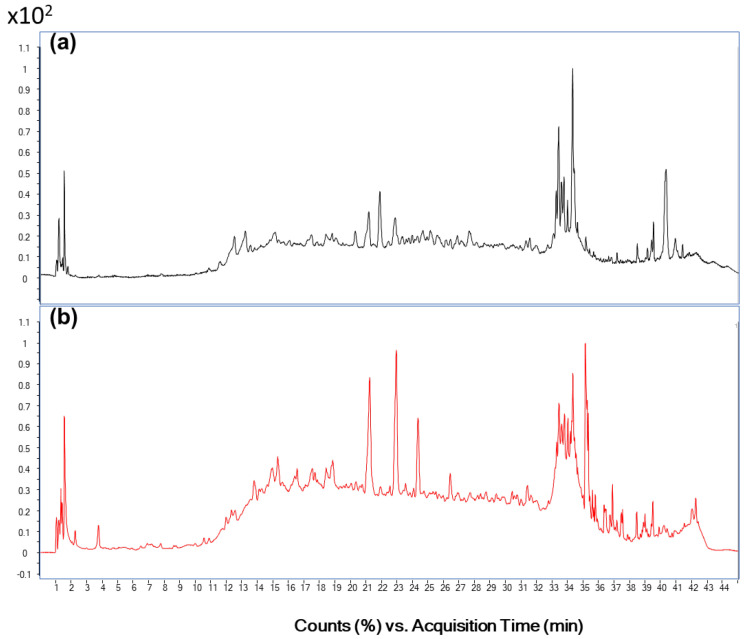
MS spectra of fraction 2, at negative (**a**) and positive (**b**) mode.

**Figure 3:**
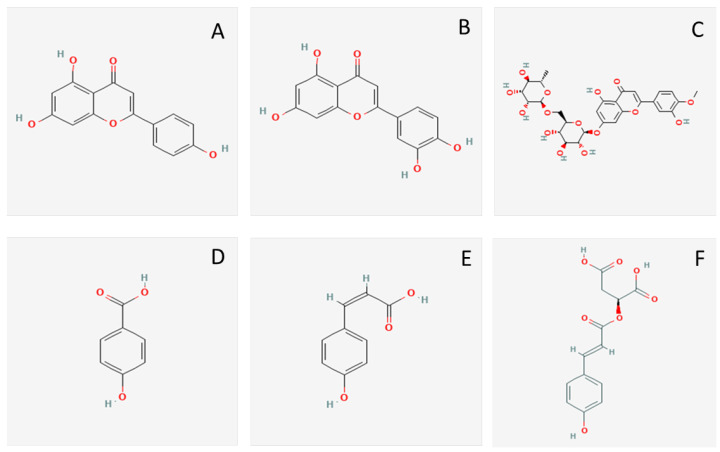
2D structures of apigenin (**A**), luteolin (**B**), diosmin (**C**), 4-hydroxybenzoic acid (**D**), coumaric acid (**E**) and 2-o(p-coumaryl)-l-malic acid (**F**) from PubChem database.

The polyphenol composition of fraction 2 was studied by LC-MS/MS (Figure 2). The MS spectra showed different peaks, but not all of them were identified as known polyphenols. The analysis of the MS/MS spectra revealed that the polyphenols present in this fraction were mainly flavones (more than 65%), of which apigenin accounted for the largest portion (38% of the total polyphenols found). Six polyphenols were identified (Table 3): 4-hydroxybenzoic acid, p-coumaric acid, a hydroxycinnamic acid derivative (2-O-(p-cumaroyl)-l-malic acid) and three flavonoids (diosmin, luteolin and apigenin). The structure of these compounds is shown in Figure 3. To our knowledge, the polyphenol composition of the ice plant has only been reported by Sánchez-Faure et al. [15]. These authors reported the presence of two hydroxycinnamic acids—one of them, hydroxybenzoic acid, also found in this study—four hydroxycinnamic acids and one flavonol. The differences in the composition may be due to different factors such as seasonality, the soil in which the plant was grown and/or the extraction method used.

The antioxidant effect of phenolic compounds has been widely reported. However, the antioxidant activity could not be attributed to one or several of the polyphenols present in the fraction, but rather to all of them together. The antioxidant activity of p-coumaric acid has been proved in vitro and in vivo and has been ascribed to its capacity to scavenge radicals and also to its metal-ion chelating ability [50]. In addition, 4-hydroxybenzoic acid has been described as an antioxidant agent, being this ability ascribed to the potential to reduce ferric ions [51]. Apigenin has been described as a free radical scavenger, with the ability to regulate the antioxidant defense in pancreatic cells. Moreover, apigenin is capable of exerting an antioxidant effect in pancreatic cells [52]. The antioxidant effect of apigenin is associated with significant anti-inflammatory effects, as reported by Ginwala et al. [53]. As well, luteolin exerts a potent antioxidant effect, mediated by the high number of OH groups in the B ring [52]. Diosmin has been reported as a potent antioxidant in in vitro and in vivo studies [54]. The antioxidant effect of the flavonoids and phenolic acids found in the extract could be attributed to the presence of hydroxyl groups in their structure, which may act as hydrogen donors and directly scavenge reactive oxygen free radicals, thus reducing their activity, as well as the ability to chelate metal ions [55]. It cannot be ruled out that the antioxidant activity was also the result of a synergistic effect of the phenolic compounds with other unidentified compounds that may be present in the fraction. The activity would then be superior to that of each of the individual components [55].

Fraction 2 showed an ACE inhibitory capacity of 100%. Polyphenols could exert an inhibitory effect on ACE through different mechanisms, according to Shukor et al. [34]. These authors studied the inhibitory effect of 22 phenolic compounds on ACE and observed that the ACE inhibitory effect of hydroxybenzoic and hydroxycinnamic acids is higher when an increased number of hydroxyl groups on the benzene ring are found. These OH groups and others such as carboxyl and acrylic acid groups can act as hydrogen bond acceptors or donors and increase the ACE-inhibiting potency. In this work, the phenolic compounds belonging to these families (4-hydroxybenzoic acid, coumaric acid and 2-O-(p-cumaroyl)-l-malic acid) presented two or three hydroxyl groups and one acrylic acid group in the molecule that could confer the ability to inhibit ACE. Although Błaszczak et al. [56] reported that p-coumaric and 4-hydroxybenzoic have a negligible effect on ACE inhibition, Men et al. [35] observed the opposite effect of 4-hydroxybenzoic acid isolated from halophyte plants. This suggests that the ACE-inhibitory effect of 4-hydroxybenzoic acid found in fraction 2 may be questioned. Diosmin, with eight hydroxyl groups in its structure, could be the main ACE-inhibitor in this fraction. Apigenin could also exert ACE-inhibiting activity, as reported by Shree et al. [57] and Loizzo et al. [58], as well as luteolin, due to the presence of the catechol group in the B-ring [59]. The results reported in the literature, therefore, indicate that the flavonoids found in fraction 2 could be the main sources responsible for the ACE-inhibitory activity, without ruling out the inhibitory effect of the phenolic acids.

Very few studies have addressed the study of PEP-inhibiting phenolic compounds from plants. Ali et al. [60] observed the PEP-inhibiting effect of quercetin isolated from Persian ironwood (*Parrotia persica*), in contrast to polyphenols isolated from hawthorn (rutin, epicatechin and chlorogenic acid) [61]. Marques et al. [7] reported potent PEP-inhibiting activity of the flavonoids oroxylin A, and mainly hispidulin and oroxyloside, from *Scutellaria racemosa*. Baicalin has also been described as a PEP inhibitor, exerting its inhibitory effect by a non-competitive mechanism [62]. According to these authors, the sugar moiety of baicalin would not be involved in the interaction with the enzyme. Similarly, Kim et al. [63] isolated some non-competitive inhibitors from green tea leaves that were identified as (−)-epigallocatechin gallate, (−)-epicatechin gallate, and (+)-gallocatechin gallate. Concerning the phenolic compounds identified in fraction 2, Fan et al. [64] reported a very low PEP-inhibiting effect of 4-hydroxybenzoic acid and p-coumaric acid extracted from the underground part of *Rhodiola sacra*. These authors reported a certain relationship between the presence of a catechol B-ring or pyrogallol groups in the molecule and the inhibition of the enzyme, which could explain the PEP inhibitory effect of the phenolic compounds present in the previously mentioned tea extract. This suggests that luteolin, with a catechol group, and diosmin, with two pyrogallol groups, could be the main PEP inhibitory polyphenols present in the fraction. In addition, the presence of a carbonyl group, as diosmin and luteolin present in the flavone skeleton, together with catechol or pyrogallol moieties, has been suggested as a structural essential feature in the inhibitory effect [65]. The PEP inhibitory activity of luteolin was previously reported by Lee et al. [66], who indicated the importance of the OH group at position 7 on the inhibitory capacity, in addition to the presence of the catechol B-ring. These authors also measured the PEP-inhibitory activity of apigenin, which was more than 50 times lower than that of luteolin. Thus, the PEP inhibitory activity of the extract seemed to be due to the presence of luteolin and diosmin.

The DPP-IV-inhibiting activity of phenolic compounds has been reported by different authors. However, to our knowledge, this is the first time that the inhibitory activity of halophyte extracts against DPP-IV has been described. Gao et al. [67] found some active sites, named S1, S2 and S3, in DPP-IV implicated in the enzymatic inhibition induced by polyphenols. These authors observed that the highest inhibitory effect was produced by isorhamnetin-3-O-glucoside, cyanidin-3-O-glucoside and isorhamnetin-3-O-rutinoside; luteolin and apigenin also showed good inhibitory activity. Fan et al. [68] analyzed the DPP-IV inhibitory effect of 27 phenolic compounds commonly present in some plants such as citrus, berries, etc.; they indicated that resveratrol, luteolin and apigenin showed the maximum inhibitory activity. The flavonoids cirsimaritin, hispidulin, naringenin, eriodictyol and rosmaric acid isolated from culinary herbs showed an efficient inhibitory effect against DPP-IV [69]. Haron et al. [70] reported a high binding affinity towards DPP-IV of robustaflavone, catechin, apigenin, kaempferol and myricetin isolated from *Anacardium occidentale*, while other polyphenols such as gallic acid, p-coumaric acid and protocatechuic acid showed lower binding affinity. Egbuna et al. [71] reviewed the antidiabetic effect of different natural compounds and described apigenin as a DPP-IV inhibitory compound. Moreover, they did describe it as an inhibitor of other enzymes involved in glucose metabolism, such as alpha-glucosidase and alpha-amylase, as well as p-coumaric acid and p-hydroxybenzoic acid as inhibitors of alpha-glucosidase. These results suggest that the DPP-IV-inhibiting activity of the isolated fraction 2 was mainly due to the presence of apigenin, p-coumaric acid and luteolin. These polyphenols account for around 61% of the compounds identified in fraction 2. To the best of our knowledge, no data had been previously reported regarding the DPP-IV inhibitory action of the other flavonoid found in this study, diosmin. Nonetheless, the DPP-IV inhibitory effect of another flavone with a rutinoside group, isorhamnetin-3-O-rutinoside, has been reported [67], suggesting that diosmin could also exert DPP-IV inhibiting activity.

## 4. Conclusions

The ice plant extract showed significant antioxidant activity and an important inhibitory effect against PEP, ACE and DPP-IV. These interesting bioactivities could be ascribed to the presence of certain polyphenols in the extract, mainly flavonoids showing inhibitory effects against some or all of the studied enzymes. Future studies are needed to determine the potential therapeutic effect of the extract, its stability during food processing (if used as an ingredient in functional foods) and bioavailability. It is also necessary to perform an in-depth study of the inhibitory effect against ACE, DPP-IV and PEP and the antioxidant effect of each isolated polyphenol, as well as to study possible synergistic effects among polyphenols and other molecules in the extract. All these studies would contribute to the upgrading of this underused and abundant halophyte.

## Figures and Tables

**Table 1:** Phenol content, antioxidant and enzymatic inhibiting activity of the ice plant extract.

Assay	*M. crystallinum* Extract
**Chemical composition**	
Total phenolic compounds (mEq GA/g)	10.02 ± 0.07
**Antioxidant activity**	
DPPH (µEq Trolox/g)	21.0 ± 0.3
ORAC (µEq Trolox/g)	88.0 ± 10.4
TEAC (µEq Trolox/g)	84.4 ± 4.6
FRAP (mEq Mohr’s salt/g)	8.9 ± 0.3
Ferrous ion chelating activity (mEq EDTA/g)	1.6 ± 0.0
**Enzymatic activity inhibition**	
ACE, 1 mg/mL (%)	90.5 ± 3.3
PEP, 1 mg/mL (%)	98.6 ± 0.1
DPP-IV, 1 mg/mL (%)	73.1 ± 3.3

**Table 2:** Phenol content, antioxidant and enzymatic inhibiting activity of the fractions obtained after chromatographic separation of the ice plant extract. Different letters in the same row indicate significant differences between pair mean values.

Assay	Fraction 1	Fraction 2
**Chemical composition**		
Total phenols (mE(q GAE/g)	9.28 ± 2.8 ^a^	61.9 ± 2.8 ^b^
**Antioxidant activity**		
DPPH (μEq Trolox/g)	64.4 ± 9.3 ^a^	185.3 ± 53.6 ^b^
ORAC (μEq Trolox/g)	427.9 ± 42.0 ^a^	1541.5 ± 78.0 ^b^
TEAC (μEq Trolox/g)	739.2 ± 88.1 ^a^	1341.7 ± 75.3 ^b^
FRAP (mEq Mohr’s salt/g)	6.4 ± 0.0 ^a^	13.5 ± 0.1 ^b^
Ferrous ion chelating activity (mEq EDTA/g)	4.8 ± 0.1 ^a^	4.4 ± 0.1 ^b^
**Enzymatic activity inhibition**		
ACE, 100 µg/mL (%)	59.3 ± 1.69 ^a^	100 ± 0.0 ^b^
PEP, 200 µg/mL (%)	0 ^a^	90.6 ± 2.5 ^b^
DPP-IV, 200 µg/mL (%)	11.7 ± 1.5 ^a^	58.7 ± 0.7 ^b^

**Table 3:** Profile of extractable polyphenolic compounds tentatively identified from the most active fraction. The relative area is referred to the total area of the compounds found.

Rt (Min)	Proposed Compound	Experimental Mass	Calculated Mass	Error (ppm)	Ms/Ms Ions	Relative Area (%)
	**Hidroxybenzoic acids**					
10.9	4-Hydroxybenzoic acid	137.0238	137.0241	−2.41	65, 69, 93, 109,119, 137	7.49
	**Hydroxycinnamic Acids**					
17.3	p-Coumaric acid	163.0401	163.0404	−3.64	65, 67, 75, 88, 119, 137, 145, 163	12.00
	**Hydroxycinnamic Acids derivatives**					
17.8	2-O-(p-Coumaroyl)-l-malic acid	279.051	279.0521	−3.38	71, 89, 115, 119, 133, 145,1 63, 189	18.60
	**Flavonoids**					
22.0	Diosmin	607.1668	607.1674	−1.60	284, 285, 299, 300, 301	17.72
28.7	Luteolin	285.0405	285.0409	−1.38	83, 107, 133, 143, 149, 151, 175, 199, 217	11.90
33.5	Apigenin	269.0455	269.0463	−2.68	107, 118, 121, 149, 151, 158, 225, 269	38.00

## Data Availability

The datasets generated for this study are available on request to the corresponding author.

## References

[1] Schmidt H., Barrett D.H., Ortmann L.W., Dawson A., Saenz C., Reis A., Bolan G. (2016). Chronic disease prevention and health promotion. Public Health Ethics: Cases Spanning the Globe.

[2] Lee J.-W., Park S.-Y., Oh M.-M. (2021). Supplemental radiation of ultraviolet-A light-emitting diode improves growth, antioxidant phenolics, and sugar alcohols of ice plant. Hortic. Environ. Biotechnol..

[3] Wijesekara I., Kim S.-K. (2010). Angiotensin-I-converting enzyme (ACE) inhibitors from marine resources: Prospects in the pharmaceutical industry. Mar. Drugs.

[4] Svarcbahs R., Julku U., Kilpeläinen T., Kyyrö M., Jäntti M., Myöhänen T.T. (2019). New tricks of prolyl oligopeptidase inhibitors—A common drug therapy for several neurodegenerative diseases. Biochem. Pharmacol..

[5] Babkova K., Korabecny J., Soukup O., Nepovimova E., Jun D., Kuca K. (2017). Prolyl oligopeptidase and its role in the organism: Attention to the most promising and clinically relevant inhibitors. Future Med. Chem..

[6] Gass J., Khosla C. (2007). Biomedicine & Diseases: Review. Prolyl endopeptidases. Cell. Mol. Life Sci..

[7] Marques M.R., Stuker C., Kichik N., Tarrago T., Giralt E., Morel A.F., Dalcol I.I. (2010). Flavonoids with prolyl oligopeptidase inhibitory activity isolated from *Scutellaria racemosa Pers*. Fitoterapia.

[8] Kazakos K. (2011). Incretin effect: GLP-1, GIP, DPP4. Diabetes Res. Clin. Pract..

[9] Singh A.-K., Yadav D., Sharma N., Jin J.-O. (2021). Dipeptidyl Peptidase (DPP)-IV inhibitors with antioxidant potential isolated from natural sources: A novel approach for the management of diabetes. Pharmaceuticals.

[10] Atzori G., de Vos A.C., van Rijsselberghe M., Vignolini P., Rozema J., Mancuso S., van Bodegom P.M. (2017). Effects of increased seawater salinity irrigation on growth and quality of the edible halophyte *Mesembryanthemum crystallinum* L. under field conditions. Agric. Water Manag..

[11] Loconsole D., Murillo-Amador B., Cristiano G., De Lucia B. (2019). Halophyte common ice plants: A future solution to arable land salinization. Sustainability.

[12] Abd El-Gawad A.M., Shehata H.S. (2014). Ecology and development of *Mesembryanthemum crystallinum L*. in the Deltaic Mediterranean coast of Egypt. Egypt. J. Basic Appl. Sci..

[13] Lee B.H., Lee C.C., Wu S.C. (2014). Ice plant (*Mesembryanthemum crystallinum*) improves hyperglycaemia and memory impairments in a Wistar rat model of streptozotocin-induced diabetes. J. Sci. Food Agric..

[14] Ibtissem B., Abdelly C., Sfar S. (2012). Antioxidant and antibacterial properties of *Mesembryanthemum crystallinum* and *Carpobrotus edulis* extracts. Adv. Chem. Eng. Sci..

[15] Sánchez-Faure A., Calvo M.M., Pérez-Jiménez J., Martín-Diana A.B., Rico D., Montero M.P., Gómez-Guillén M.C., López-Caballero M.E., Martínez-Alvarez O. (2020). Exploring the potential of common iceplant, seaside arrowgrass and sea fennel as edible halophytic plants. Food Res. Int..

[16] Brand-Williams W., Cuvelier M.E., Berset C. (1995). Use of a free radical method to evaluate antioxidant activity. LWT-Food Sci. Technol..

[17] Ou B., Hampsch-Woodill M., Prior R.L. (2001). Development and validation of an improved oxygen radical absorbance capacity assay using fluorescein as the fluorescent probe. J. Agric. Food Chem..

[18] Re R., Pellegrini N., Proteggente A., Pannala A., Yang M., Rice-Evans C. (1999). Antioxidant activity applying an improved ABTS radical cation decolorization assay. Free Radic. Biol. Med..

[19] Benzie I.F.F., Strain J.J. (1996). The ferric reducing ability of plasma as a measure of “antioxidant power”: The FRAP assay. Anal. Biochem..

[20] Sentandreu M.A., Toldrá F.A. (2006). Rapid, simple and sensitive fluorescence method for the assay of angiotensin-I converting enzyme. Food Chem..

[21] Sila A., Martinez-Alvarez O., Haddar A., Gómez-Guillén M.C., Nasri M., Montero M.P., Bougatef A. (2015). Recovery, viscoelastic and functional properties of Barbel skin gelatine: Investigation of anti-DPP-IV and anti-prolyl endopeptidase activities of generated gelatine polypeptide. Food Chem..

[22] Lucas-Abellán C., Mercader-Ros M.T., Zafrilla M.P., Gabaldón J.A., Núñez-Delicado E. (2011). Comparative study of different methods to measure antioxidant activity of resveratrol in the presence of cyclodextrins. Food Chem. Toxicol..

[23] Huang D., Ou B., Prior R.L. (2005). The chemistry behind antioxidant capacity assays. J. Agric. Food Chem..

[24] Ngxabi S., Jimoh M.O., Kambizi L., Laubscher C.P. (2021). Growth characteristics, phytochemical contents, and antioxidant capacity of *Trachyandra ciliata* (L.f) Kunth grown in hydroponics under varying degrees of salinity. Horticulturae.

[25] Souid A., Croce C.M.D., Frassinetti S., Gabriele M., Pozzo L., Ciardi M., Abdelly C., Hamed K.B., Magné C., Longo V. (2021). Nutraceutical potential of leaf hydro-ethanolic extract of the edible halophyte *Crithmum maritimum* L.. Molecules.

[26] Boestfleisch C., Papenbrock J. (2017). Changes in secondary metabolites in the halophytic putative crop species *Crithmum maritimum L.*, *Triglochin maritima L.* and *Halimione portulacoides* (L.) Aellen as reaction to mild salinity. PLoS ONE.

[27] Cybulska I., Zembrzuska J., Brudecki G., Thomsen M.H. (2021). Optimizing methods to characterize caffeic, ferulic, and chlorogenic acids in *Salicornia sinus-persica* and *Salicornia bigelovii* extracts by tandem mass spectrometry (LC-MS/MS). Bioresources.

[28] Hanen F., Riadh K., Samia O., Sylvain G., Christian M., Chedly A. (2009). Interspecific variability of antioxidant activities and phenolic composition in Mesembryanthemum genus. Food Chem. Toxicol..

[29] Ksouri R., Ksouri W.M., Jallali I., Debez A., Magné C., Hiroko I., Abdelly C. (2012). Medicinal halophytes: Potent source of health promoting biomolecules with medical, nutraceutical and food applications. Crit. Rev. Biotechnol..

[30] Lin Y.H., Lin Y.K., Lin Y.H. (2019). Photoprotective effects of ice plant (*Mesembryanthemum crystallinum*) callus extract on gene expression of human dermal fibroblast against UV exposure. J. Biobased Mater. Bioenergy.

[31] Seo J.A., Ju J. (2019). Antioxidant and growth inhibitory activities of *Mesembryanthemum crystallinum* L. in HCT116 human colon cancer cells. J. Nutr. Health.

[32] Qasim M., Abideen Z., Adnan M.Y., Gulzar S., Gul B., Rasheed M., Khan M.A. (2017). Antioxidant properties, phenolic composition, bioactive compounds and nutritive value of medicinal halophytes commonly used as herbal teas. S. Afr. J. Bot..

[33] Sadeer N.B., Sinan K.I., Cziaky Z., Jeko J., Zengin G., Jeewon R., Abdallah H.H., Rengasamy K.R.R., Mahomoodally M.F. (2020). Assessment of the pharmacological properties and phytochemical profile of *Bruguiera gymnorhiza* (L.) lam using in vitro studies, in silico docking, and multivariate analysis. Biomolecules.

[34] Shukor N.A., Van Camp J., Gonzales G.B., Staljanssens D., Struijs K., Zotti M.J., Raes K., Smagghe G. (2013). Angiotensin-converting enzyme inhibitory effects by plant phenolic compounds: A study of structure activity relationships. J. Agric. Food Chem..

[35] Men R., Li N., Xing Y., Tang Y., Tan C., Meng F., Zhang J., Ni H., Ji X. (2013). Chemical constituents and ACE inhibitory activity of desert plant *Suaeda physophora* Pall. Acta Pharm. Sin. B.

[36] Cho J.Y., Park K.H., Hwang D.Y., Chanmuang S., Jaiswal L., Park Y.K., Park S.Y., Kim S.Y., Kim H.R., Moon J.H. (2015). Antihypertensive effects of *Artemisia scoparia waldst* in spontaneously hypertensive rats and identification of angiotensin I converting enzyme inhibitors. Molecules.

[37] Oliveira-Alves S.C., Andrade F., Prazeres I., Silva A.B., Capelo J., Duarte B., Cacador I., Coelho J., Serra A.T., Bronze M.R. (2021). Impact of drying processes on the nutritional composition, volatile profile, phytochemical content and bioactivity of *Salicornia ramosissima* J. woods. Antioxidants.

[38] Sharifi A.M., Darabi R., Akbarloo N. (2003). Study of antihypertensive mechanism of *Tribulus terrestris* in 2K1C hypertensive rats: Role of tissue ACE activity. Life Sci..

[39] Phillips O.A., Mathew K.T., Oriowo M.A. (2006). Antihypertensive and vasodilator effects of methanolic and aqueous extracts of *Tribulus terrestris* in rats. J. Ethnopharmacol..

[40] Gattringer J., Ndogo O.E., Retzl B., Ebermann C., Gruber C.W., Hellinger R. (2021). Cyclotides isolated from Violet plants of Cameroon are inhibitors of human Prolyl Oligopeptidase. Front. Pharmacol..

[41] Tarrago T., Kichik N., Segui J., Giralt E. (2007). The natural product berberine is a human prolyl oligopeptidase inhibitor. Chem. Med. Chem..

[42] Benwahhoud M., Jouad H., Eddouks M., Lyoussi B. (2001). Hypoglycemic effect of *Suaeda fruticosa* in streptozotocin-induced diabetic rats. J. Ethnopharmacol..

[43] Farias D.D., de Araujo F.F., Neri-Numa I.A., Pastore G.M. (2021). Antidiabetic potential of dietary polyphenols: A mechanistic review. Food Res. Int..

[44] Ansari M.H.R., Ahmad S. (2019). Herbs that heal: Natural remedies for health promotion and longevity. Ann. Phytomed. Int. J..

[45] Calvo M.M., Tzamourani A., Martínez-Alvarez O. (2021). Halophytes as a potential source of melanosis-inhibiting compounds. Mechanism of inhibition of a characterized polyphenol extract of purslane (*Portulaca oleracea*). Food Chem..

[46] Aleixandre-Tudo J.L., Buica A., Nieuwoudt H., Aleixandre J.L., du Toit W. (2017). Spectrophotometric analysis of phenolic compounds in grapes and wines. J. Agric. Food Chem..

[47] Cianciosi D., Forbes-Hernandez T.Y., Regolo L., Alvarez-Suarez J.M., Navarro-Hortal M.D., Xiao J.B., Quiles J.L., Battino M., Giampieri F. (2022). The reciprocal interaction between polyphenols and other dietary compounds: Impact on bioavailability, antioxidant capacity and other physico-chemical and nutritional parameters. Food Chem..

[48] Slika H., Mansour H., Wehbe N., Nasser S.A., Iratni R., Nasrallah G., Shaito A., Ghaddar T., Kobeissy F., Eid A.H. (2022). Therapeutic potential of flavonoids in cancer: ROS-mediated mechanisms. Biomed. Pharmacother..

[49] Li Y., Jiang H., Huang G. (2017). Protein hydrolysates as promoters of non-haem iron absorption. Nutrients.

[50] Shen Y., Song X., Li L., Sun J., Jaiswal Y., Huang J., Liu C., Yang W., Williams L., Zhang H. (2019). Protective effects of p-coumaric acid against oxidant and hyperlipidemia-an in vitro and in vivo evaluation. Biomed. Pharmacother..

[51] Manuja R., Sachdeva S., Jain A., Chaudhary J. (2013). A comprehensive review on biological activities of p-hydroxy benzoic acid and its derivatives. Int. J. Pharm. Sci. Rev. Res..

[52] Shen N., Wang T., Gan Q., Liu S., Wang L., Jin B. (2022). Plant flavonoids: Classification, distribution, biosynthesis, and antioxidant activity. Food Chem..

[53] Ginwala R., Bhavsar R., Chigbu D.G.I., Jain P., Khan Z.K. (2019). Potential role of flavonoids in treating chronic inflammatory diseases with a special focus on the anti-inflammatory activity of apigenin. Antioxidants.

[54] Gerges S.H., Wahdan S.A., Elsherbiny D.A., El-Demerdash E. (2022). Pharmacology of diosmin, a citrus flavone glycoside: An updated review. Eur. J. Drug Metab. Pharmacokinet..

[55] Lv Q.Z., Long J.T., Gong Z.F., Nong K.Y., Liang X.M., Qin T., Huang W., Yang L. (2021). Current state of knowledge on the antioxidant effects and mechanisms of action of polyphenolic compounds. Nat. Prod. Commun..

[56] Błaszczaka W., Latochab P., Jeża M., Wiczkowskia W. (2021). The impact of high-pressure processing on the polyphenol profile and anti-glycaemic, anti-hypertensive and anti-cholinergic activities of extracts obtained from kiwiberry (*Actinidia arguta*) fruits. Food Chem..

[57] Shree V.S., Sathishkumar T., Kumaresan K., Rapheal V.S., Muthukumaran P., Muthukumaran V. (2021). Therapeutic effects of purified polyphenols from *Coccinia grandis*: Correlation between hypertension and diabetes mellitus. Adv. Tradit. Med..

[58] Loizzo M.R., Said A., Tundis R., Rashed K., Statti G.A., Hufner A., Menichini F. (2007). Inhibition of angiotensin converting enzyme (ACE) by flavonoids isolated from *Ailanthus excelsa* (Roxb) (Simaroubaceae). Phytother. Res..

[59] Guerrero L., Castillo J., Quinones M., Garcia-Vallve S., Arola L., Pujadas G., Muguerza B. (2012). Inhibition of angiotensin-converting enzyme activity by flavonoids: Structure-activity relationship studies. PLoS ONE.

[60] Ali H., Ullah K., Siddiqui H., Iqbal S., Wahab A., Goren N., Ayatollahi S.A., Rahman A., Choudhary M.I. (2020). Chemical constituents from *Parrotia persica*- Structural derivatization and their potential prolyl endopeptidase inhibition activity. Bioorg. Chem..

[61] Cui T., Nakamura K., Tian S., Kayahara H., Tian Y.L. (2006). Polyphenolic content and physiological activities of Chinese hawthorn extracts. Biosci. Biotechnol. Biochem..

[62] Tarragó T., Kichik N., Claasen B., Prades R., Teixidó M., Giralt E. (2008). Baicalin, a prodrug able to reach the CNS, is a prolyl oligopeptidase inhibitor. Bioorg. Med. Chem..

[63] Kim J.-H., Kim S.-I., Song K.-S. (2001). Prolyl Endopeptidase inhibitors from green tea. Arch. Pharm. Res..

[64] Fan W.Z., Tezuka Y., Komatsu K., Namba T., Kadota S. (1999). Prolyl endopeptidase inhibitors from the underground part of *Rhodiola sacra S.H*. Fu. Biol. Pharm. Bull..

[65] Lee S.-H., Jun M., Choi J.-Y., Yang E.-J., Hur J.-M., Bae K., Seong Y.-H., Huh T.-L., Song K.-S. (2007). Plant phenolics as Prolyl Endopeptidase inhibitors. Arch. Pharm. Res..

[66] Lee K.-H., Kwak J.H., Lee K.-B., Song K.-S. (1998). Prolyl Endopeptidase inhibitors from *Caryophylli Flos*. Arch. Pharm. Res..

[67] Gao F., Fu Y., Yi J., Gao A., Jia Y., Cai S. (2020). Effects of different dietary flavonoids on Dipeptidyl Peptidase-IV activity and expression: Insights into structure-activity relationship. J. Agric. Food Chem..

[68] Fan J., Johnson M.H., Lila M.A., Yousef G., Gonzalez de Mejia E. (2013). Berry and citrus phenolic compounds inhibit Dipeptidyl Peptidase IV: Implications in diabetes management. Evid. Based Complement. Altern. Med..

[69] Bower A.M., Real Hernandez L.M., Berhow M.A., Gonzalez de Mejia E. (2014). Bioactive compounds from culinary herbs inhibit a molecular target for type 2 diabetes management, dipeptidyl peptidase IV. J. Agric. Food Chem..

[70] Haron N., Nadia N., Farahin P.N., Susanti D., Hasniza N., Bariyya K., Halim A. (2021). Molecular docking of polyphenol compounds from *Anacardium occidentale* with alpha-glucosidase and dipeptidyl-peptidase-4 enzymes. Malays. J. Fundam. Appl. Sci..

[71] Egbuna C., Awuchi C.G., Kushwaha G., Rudrapal M., Patrick-Iwuanyanwu K.C., Singh O., Odoh U.E., Khan J., Jeevanandam J., Kumarasamy S. (2021). Bioactive compounds effective against type 2 Diabetes Mellitus: A systematic review. Curr. Top. Med. Chem..

